# Supplementary material for: Electrochemical Degradation of Sulfamethoxazole Enhanced by Bio-Inspired Iron-Nickel Encapsulated Biochar Particle Electrode
Source: Int J Mol Sci. 2024 Dec 19;25(24):13579. doi: 10.3390/ijms252413579 (PMC11678343; doi:10.3390/ijms252413579)
Supplement: Supplementary file 1 [file ijms-25-13579-s001.zip › ijms-3369389-supplementary.pdf]

Bio-inspired iron-nickel encapsulated biochar particle electrode enables enhanced electrochemical degradation of sulfamethoxazole

Shuang Geng[a,c], Jingang Yao[a,b,*], Lei Wang[c,*], Yangyang Wang[c,d,*], Xiaoshu Wang[c], Junmin Li[a,c]

[a] *School of Agricultural Engineering and Food Science, Shandong University of Technology, Zibo 255000, PR China;*

[b] *School of Environmental Science and Engineering, Tianjin University, Tianjin 300072, China*

[c] *School of Materials and Environmental Engineering, Institute of Urban Ecology and Environment Technology, Shenzhen Polytechnic University, Shenzhen 518055, China;*

[d] *School of Resource and Environmental Engineering, Wuhan University of Technology, Wuhan 430070, P. R. China.*

[*] Corresponding author. *Email address:* yaojingang@tju.edu.cn (J. Yao), wlwanglei@szpu.edu.cn (L. Wang).

Text S1

Wetland emergent plants and hydroponic culture

Iris sibirica L., a common wetland emergent plant with substantial biomass, was utilized in this study. Six-month-old plants of equal weight were transplanted into a greenhouse located in the Shenzhen district, Guangdong. The sediment on the plant surfaces was thoroughly removed using tap water followed by repeated rinsing with deionized water. After washing and weighing, the plants were cultured in polyvinyl chloride (PVC) buckets (20 cm in diameter, 30 cm in length). Each bucket contained three plants and 10 liters of nutrient hydroponic solution.

The plants were exposed to nutrient solutions with varying concentrations of iron ions (0, 300 and 500 mg^{-1}) and nickel ions (0, 300 and 500 mg^{-1}). To ensure experimental accuracy, triplicate tests were conducted for each concentration of iron and nickel ions. Deionized water was added daily to maintain a constant solution volume compensating for evaporation. The nutrient solutions were completely replaced weekly. The plants were cultured for 60 days before harvesting (from September 1, 2022, to November 31, 2022). The greenhouse temperature ranged from 18 °C to 22 °C with an average photoperiod of 11/13 hours (light/dark).

The nutrient solution for culturing plants included $Ca(NO_3)_2 \cdot 4H_2O$ (5 mM), KH_2PO_4 (1 mM), KNO_3 (5 mM), $MgSO_4 \cdot 7H_2O$ (2 mM), $MnCl_2 \cdot 4H_2O$ (9 μM), $ZnSO_4 \cdot 7H_2O$ (0.8 μM), $CuSO_4 \cdot 5H_2O$ (0.3 μM), H_3BO_3 (0.05 mM), $H_2MoO_4 \cdot H_2O$ (0.1 μM) and EDTA-Fe (0.04 mM).

Text S2

Wetland emergent plants and hydroponic culture

Sample collection and catalysts preparation

Sample collection. After 60 days of hydroponic culture, plant samples were collected and washed with 0.01 M HCl and then separated into roots and shoots in the laboratory. Each sample was oven-dried at 70 °C for 72 hours, ground into a fine powder using an analytical mill and homogenized to ensure uniform element distribution. The powder was passed through a 160 μm sieve, weighed and prepared for subsequent analysis.

Bio-inspired iron-nickel encapsulated biochar (FeNi@BC) preparation. The bio-inspired FeNi@BC sample was obtained by pyrolysis at 500 °C, 700 °C and 900 °C for 2 hours in a tube muffle furnace (SK-G08163-3) under nitrogen condition.

Synthetic iron-nickel laden biochar (FeNi-BC) preparation. 3.10 g BC sample was added to 100.00 mL of a solution containing $FeCl_2 \cdot 4H_2O$ and $NiCl_2 \cdot 6H_2O$. The concentration of Fe and Ni ions was set to match that of FeNi5@BC9. The mixture was soaked at room temperature for 24 hours and then transferred to a rotary evaporator where the water was completely evaporated. The evaporated samples were placed in an oven at 80 °C for 24 hours. FeNi-BC samples were obtained through pyrolysis in a tube furnace at 900 °C for 2 hours under a nitrogen atmosphere.

Sample pretreatment. The obtained biochar samples were washed with ethanol three times and then washed repeatedly with water until the pH of the supernatant reached approximately 7.0. Afterward, all samples were dried at 60 °C for 72 hours and sealed at

4 °C in a refrigerator.

Text S3

The operating parameters of the electrochemical workstation

The electrochemical properties of the obtained samples were determined using a conventional three-electrode system with a CHI 660E electrochemical workstation. The prepared samples, a platinum sheet and a saturated calomel electrode (SCE) served as the working, counter and reference electrodes, respectively, while 0.1 mol^{-1} sodium sulfate solution was used as the electrolyte.

The prepared material was thoroughly ground using an onyx mortar and pestle. 5 mg sample was weighed and added to a 3 mL centrifuge tube containing 20 μL of 5% Nafion® 117 solution and 1 mL of isopropanol, then ultrasonically dispersed for 10-40 minutes to form a homogeneously dispersed ink slurry. Subsequently, 20 μL of the dispersion was measured and added dropwise to the conductive circular area of the glassy carbon electrodes using a pipette. After drying at room temperature, this dropwise addition was repeated two more times. Electrochemical testing was performed after complete drying. The three electrodes were then placed in 0.1 mol^{-1} sodium sulfate solution for subsequent electrochemical tests.

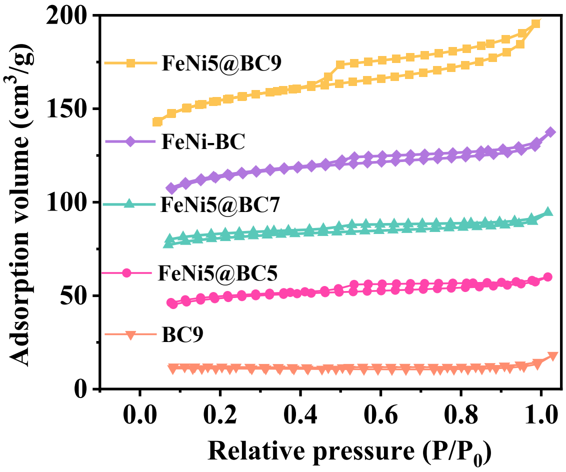

Fig. S1 N_2 adsorption-desorption isotherm of BC, FeNi-BC and FeNi@BC.

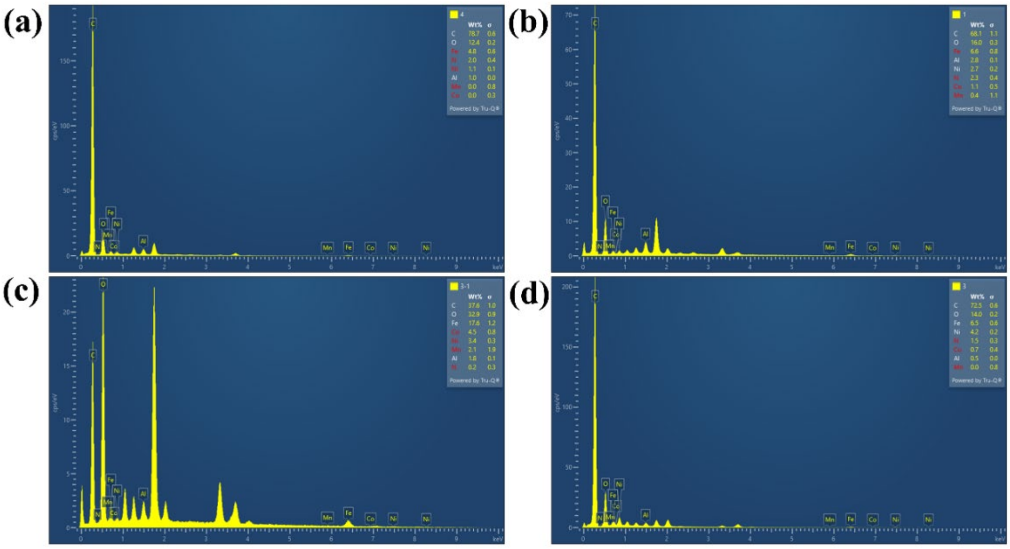

Fig. S2 EDS images of FeNi5@BC5 (a), FeNi5@BC7 (b), FeNi5@BC9 (c) and FeNi-BC5.

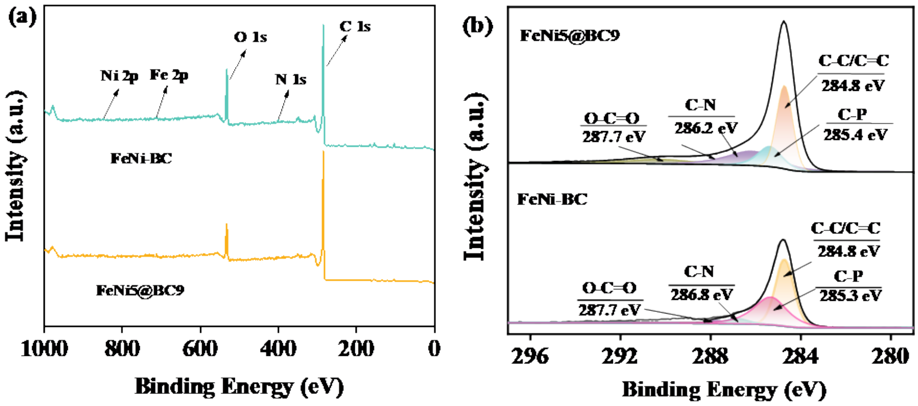

Fig. S3 XPS full spectra (a) and C1s (b) of FeNi5@BC9 and FeNi-BC.

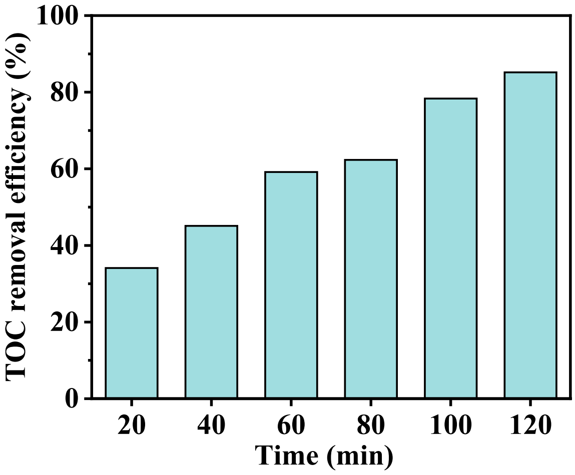

Fig. S4 The TOC removal efficiency of SMX before and after degradation (pH = 3, voltage = 8 V, the dose of catalyst = 10 mg, $[SMX]_0$ = 20 mg L^{-1}, Na_2SO_4 dose = 0.10 M and the degradation time = 120 min).

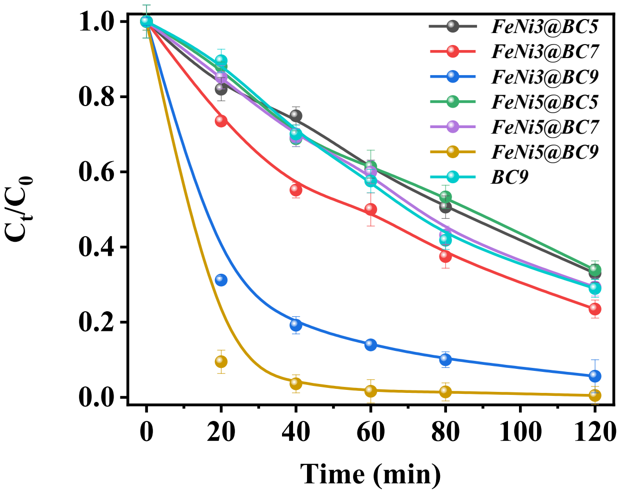

Fig. S5 Degradation performance of catalysts with different metal concentrations and pyrolysis temperatures for SMX.

Table. S1 The structural information of the possible intermediate products.

Compound	Molecular structure	Mass/charge ratio (m/z)
SMX	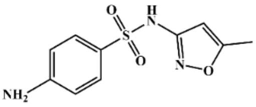	254
P1-1	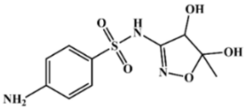	288
P1-2	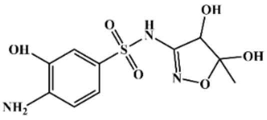	304
P1-3	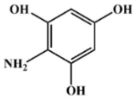	142
P1-4	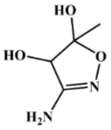	131
P2-1	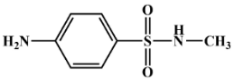	187
P2-2	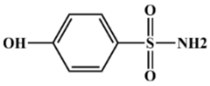	173
P2-3	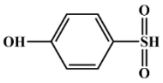	159
P3-1	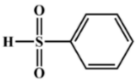	142
P3-2	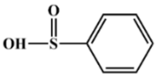	142
P3-3	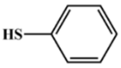	110
P4-1	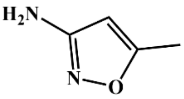	97
P4-2	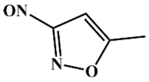	111
